# The Relationship between the Neighborhood Safety and Nutritional Status of Children in Baghdad City, Iraq

**DOI:** 10.1155/2014/686934

**Published:** 2014-08-24

**Authors:** Hasanain Faisal Ghazi, Zaleha Md. Isa, Shamsul Azhar Shah, Mohammed A. Abdal Qader, Tiba Nezar Hasan, AL-abed Ali AL-abed

**Affiliations:** Department of Community Health, Universiti Kebangsaan Malaysia Medical Centre, Bandar Tun Razak, Cheras, 56000 Kuala Lumpur, Malaysia

## Abstract

*Introduction.* The neighborhoods where the children live play an important role in their development physically and mentally. The objective of this study was to assess the relationship between neighborhood safety and child nutritional status in Baghdad city, Iraq. *Methods.* A cross-sectional study was carried out in Baghdad city, Iraq, among 400 primary school children from 4 schools. BMI-for-age *Z* score was used to assess the nutritional status of the children. Newly developed questionnaires on neighborhood safety were distributed to the parents to answer them. *Results.* In this study, males were more predominant than females with 215 participants compared to 185 females. A total of 49% were normal weight, 38.8% either overweight or obese, and only 12.2% underweight. There was a significant relationship between father education, father and mother working status, family income, and children nutritional status (*P* = 0.10, 0.009, <0.001, 0.37), respectively. The association between neighborhood safety variables and child nutritional status was significant except for worrying about child safety and thinking of leaving the neighborhood (*P* = 0.082, 0.084), respectively.* Conclusion.* Nutritional status of school children continues to be a public health issue in Iraq especially Baghdad city. There was a significant association between neighborhood safety and children nutritional status.

## 1. Introduction

Child nutrition remains a serious challenging issue not only in Iraq but also worldwide. As a developing country located in the Middle East, Iraq faced several conflicts starting from 1980 followed by 1991 war then 13 years of economic sanctions and last war in 2003. The problem for Iraqis and especially children is not the war itself but the following years of unstable situation that can impact their daily life and activities.

Environmental factors can include various physical and social elements in people's surroundings that influence their choices for diet and physical activities. School and nearby neighborhood environments are of interest because children spend at least 5–7 hours daily within school boundaries during weekdays and make various health choices while at school [1].

Iraq is a developing country experiencing constraints on economic and social development and most of the environmental factors that affect the physical growth of children before puberty, including poor food consumption patterns, illness, lack of sanitation, poor hygiene practices, and poor health care coverage and resources, are present. Almost half of Iraq's total populations of 27 million are children. United Nations agencies estimate that 1 out of 8 Iraqi children dies before the age of 5 years; one-third are undernourished; one-quarter are born underweight and one-quarter do not have access to safe water. Rates for stunting among children under the age of 5 years in southern and central Iraq peaked in 1996 at 32.0% and then declined to 23.1% in 2002 [2].

ALDoori et al. [3] did a study in Basrah city in south of Iraq following the 1991 war and found that 24% of the children were stunted. Stunting and low weight-for-age were significantly higher among children of low socioeconomic households. Comparison of these data with an earlier nutritional survey in the area showed the nutritional status of children in Basrah city has deteriorated as a result of successive armed conflicts.

Another study done by Guerrero-serdan [4] in Iraq assumed the war has affected the physical growth of children, where the young born after the war in high-intensity conflict areas had lower height-for-age* Z*-scores than children born in less violent areas. Also the weight-forage* Z*-scores increased in 2004 but decreased in 2006. These results suggest that children are not losing weight but rather are not growing properly in length in high violence affected areas.

The links between neighborhood and nutritional status can be because of many reasons such as safety concerns (crime; unstable political situation), the environment (lack of parks, playgrounds, and walkable destinations such as restaurants and supermarkets), and access to and affordability of healthy foods [5–9].

The objective of this study was to assess the effects of unstable neighborhood in the last 8 years in Baghdad city on child nutritional status.

## 2. Methods

A cross-sectional study was conducted among 400 respondents aged between 7 and 8 years from four different primary schools in Baghdad city in 2011. Baghdad is divided into two sides by Tigris river, so 2 schools from each side were chosen randomly. Subsequently, a complete list of student names in each selected school was obtained. A total of 100 children from each school were then identified by stratified random sampling according to grade. Questionnaires on neighborhood safety and standardized measurements for weight and height were used in this study. Self-administered questionnaires were distributed to respondents' parents during the monthly meeting at schools.

BMI for age* Z*-score was used to assess the nutritional status of the children based on WHO 2007 growth references cut-off points [10]. Weight and height of the respondents were measured by using calibrated weighing machine to be compared to WHO cut-off points. Weight was measured using digital weighing scale manufactured by Beurer Company in Germany. Children were weighed after taking off their shoes.

Height was measured by using tape measure to the nearest 0.5 cm by standing with their back touching the wall and their head in upright position. Then BMI-for-Age* Z* score is recategorized into three groups, underweight, normal weight, and overweight or obese children in one group.

Neighborhood safety in this study was defined as the place where the child's family is living. The questionnaire consists of 10 items, each with three response options: never (2), sometimes (1), and a lot of times (0). The maximum total score was 20. A score of 10 was taken as the cut-point because if the respondents were to answer “sometimes” for all questions, the total score is 10. Any score less than 10 indicated unsafe neighborhood, whereas a score equal to or above 10 indicated safe neighborhood.

A questionnaire validation was carried out using face validity, factor analysis, and back-to-back translation. After that a pretest was done by distributing the questionnaires to 50 parents to ensure that the questions are easily understandable and validated. Living environment scale has a good internal consistency, with Cronbach's alpha coefficient of 0.820.

This study was approved by the Research and Ethics Committee of Universiti Kebangsaan Malaysia Medical Center.

## 3. Results

The response rate in this study was 85.1% (400/470). The results show that the mean body weight of the children was 25.20 ± 5.6 kg. The average standing height of the children was 121.00 ± 8.8 cm. The most common nutritional status was normal (49.0%) followed by overweight and obese (38.8%) and underweight (12.2%).

There was a significant relationship between father education, father and mother working status, family income, and children nutritional status (*P* = 0.10, 0.009, <0.001, 0.37), respectively, as shown in Table 1.

For neighborhood safety variables, the relationship between thinking of unstable situation, hearing gun shots, hearing explosions, saying dead bodies, child witnessing explosions, difficulties in going to school, thinking neighborhood is unsafe, child missing school days, and nutritional status was significant (*P* = 0.034, <0.001, 0.023, <0.001, 0.024, <0.001, 0.030, <0.001), respectively, as shown in Table 2.

There was a significant association between neighborhood safety status and nutritional status of the children (*P* < 0.001) as shown in Table 3, which means children living in unsafe neighborhood have more risk to be overweight or obese as they tend to be physically inactive.

## 4. Discussion

In this study, the neighborhood safety status reflects the daily life of Iraqi families and their children. There was some improvement in the living environment due to changes in the security situation in the capital Baghdad in the last 2 years as more than two-thirds of the children's parents were found to live in a good environment according to our cut-off point.

The nutritional status of the children was affected by the unstable living environment in Baghdad city in the last 10 years after the end of 2003 war. The association between neighborhood safety status and child nutritional status was significant, which means children who live in bad living environment have more risk to be overweight and obese compared to those living in acceptable living environment and this may be due to lack of physical activity as the children cannot play outside the house in the playground and also cannot walk to school because parents are concerned about their safety.

Tol et al. [11] noted that exposure to violence is a risk factor for adverse outcomes of child development in low-income settings, and that childhood mental health problems are difficult to address within the contexts of ongoing poverty and political instability. Parental perception of neighborhood safety likely operates more strongly than that of the child in altering lifestyle because parents exert significant control over young children's activities [12, 13].

The results of this study was supported by similar studies done in armed conflicts zones elsewhere in the world, such as one study done following the conflict in Guinea-Bissau [14]. They found that in a noncamp setting, residents may be more malnourished and have higher mortality than refugees. Major improvements in nutritional status and a reduction in mortality occurred in resident and refugee children as soon as refugees returned home despite the fact that there was no improvement in food availability.

Previous study done in Basrah city in south of Iraq following the 1991 war [3] found that 24% of the children were stunted. Stunting and low weight-for-age were significantly higher among children of low socioeconomic households. Comparison of these data with an earlier nutritional survey in the area showed the nutritional status of children in Basrah city has worsened as a result of successive armed conflicts.

Latest study done to assess the effects of the 2003 war in Iraq on nutrition and health [4] concluded that the war had affected the physical growth of children, where young cohorts born after the war in high-intensity conflict areas had lower height-for-age* Z*-scores than children born in less violent areas. Also the weight-forage* Z*-scores increased in 2004 but decreased in 2006. These results suggest that children are not losing weight but rather are not growing properly in length in high violence affected areas.

There were some limitations in this study. First of all, its cross-sectional design only measured the prevalence at specific point of time in specified areas. Second, is the recall bias of the respondents' parents, especially in their evaluation of the security situation in their living neighborhood. lastly, is the logistic issues because of unstable security situation.

## 5. Conclusion

Nutritional status of school children continues to be a public health issue in Iraq especially Baghdad city. There was a significant association between neighborhood safety and children nutritional status. There is a transition from undernutrition among children during the economic sanctions in the 90s to rapid increase in overweight and obesity prevalence in Iraq. Children living in safe area tend to be more overweight and obese.

## Figures and Tables

**Table 1:** Sociodemographic characteristics of children.

Variables	Nutritional status			*P* value
	Underweight *N* (%)	Normal *N* (%)	Overweight and obese *N* (%)	
Gender				
Male	23 (10.7)	109 (50.7)	83 (38.6)	0.551^a^
Female	26 (14.1)	87 (47.0)	72 (38.9)	
Mother education				
Low	10 (16.9)	28 (47.5)	21 (35.6)	0.448^a^
High	39 (11.5)	166 (49.0)	134 (39.5)	
Father education				
Low	4 (8.7)	32 (69.6)	10 (21.7)	0.010^a^
High	36 (10.7)	155 (46.3)	144 (43.0)	
Mother working status				
Housewife	35 (16.8)	101 (48.6)	72 (34.6)	0.009^a^
Working	14 (7.4)	93 (48.9)	83 (43.7)	
Father working status				
Not working	6 (42.9)	6 (42.9)	2 (14.2)	<**0**.001^a^
Working	34 (9.3)	181 (49.3)	152 (41.4)	
Family income				
Mean ± SD	615,673.46 ± 361,872.29	763,418.36 ± 546,916.34	810,225.80 ± 356,521.04	0.037^b^

^a^Chi Square test was performed; ^b^Student *t*-test was performed, level of significance at *P* value <0.05.

**Table 2:** Relationship between neighbourhood safety variables and children nutritional status.

Variables	Nutritional status			*P* value^a^
	Underweight *N* (%)	Normal *N* (%)	Overweight and obese *N* (%)	
Did that unstable security situation affect your daily life?				
A lot of times	15 (19.7)	30 (39.5)	31 (40.8)	**0.034**
Sometimes	29 (10.8)	131 (48.7)	109 (40.5)	
Never	5 (9.1)	35 (63.6)	15 (27.3)	
Did you hear gun shots in your neighborhood during the last month?				
A lot of times	8 (13.1)	26 (42.6)	27 (44.3)	<**0.001**
Sometimes	37 (14.0)	146 (55.3)	81 (30.7)	
Never	4 (5.3)	24 (32.0)	47 (62.7)	
Did you hear explosions in your neighborhood during the last month?				
A lot of times	8 (13.1)	29 (47.5)	24 (39.4)	**0.023**
Sometimes	38 (15.2)	125 (50.0)	87 (34.8)	
Never	3 (3.4)	42 (47.2)	44 (49.4)	
Did you see any dead bodies in your neighborhood during the last month?				
A lot of times	2 (33.3)	4 (66.7)	0 (0.0)	<**0.001**
Sometimes	27 (30.7)	37 (42.0)	24 (27.3)	
Never	20 (6.5)	155 (50.7)	131 (42.8)	
Did your child witness any explosions in your neighborhood?				
A lot of times	4 (40.0)	4 (40.0)	2 (20.0)	**0.024**
Sometimes	27 (14.8)	84 (45.9)	72 (39.3)	
Never	18 (8.7)	108 (52.2)	81 (39.1)	
Did your child face any problems like closed roads in going to school during the last month?				
A lot of times	12 (54.5)	6 (27.3)	4 (18.2)	<**0.001**
Sometimes	25 (13.2)	82 (43.1)	83 (43.7)	
Never	12 (6.4)	108 (57.4)	68 (36.2)	
Do you worry about child safety when they go to school?				
A lot of times	23 (18.4)	57 (45.6)	45 (36.0)	0.082
Sometimes	17 (8.5)	99 (49.2)	85 (42.3)	
Never	9 (12.2)	40 (54.0)	25 (33.8)	
Did your child miss any school days because of neighborhood safety issues?				
A lot of times	6 (37.4)	5 (31.3)	5 (31.3)	**0.030**
Sometimes	27 (11.0)	119 (48.4)	100 (40.6)	
Never	16 (11.6)	72 (52.2)	50 (36.2)	
Do you ever think of leaving your neighborhood?				
A lot of times	9 (20.5)	22 (50.0)	13 (29.5)	0.084
Sometimes	26 (12.7)	90 (44.1)	88 (43.2)	
Never	14 (9.2)	84 (55.3)	54 (35.5)	
Do you think that your neighborhood area is insecure?				
A lot of times	10 (37.0)	10 (37.0)	7 (26.0)	<**0.001**
Sometimes	21 (10.1)	88 (42.3)	99 (47.6)	
Never	18 (10.9)	98 (59.4)	49 (29.7)	

^a^Pearson Chi Square test was performed; level of significance is at *P* < 0.05.

**Table 3:** The relationship between neighborhood safety status and children nutritional status.

Neighborhood safety	Nutritional status			*P* value
	Underweight *N* (%)	Normal *N* (%)	Overweight and obese *N* (%)	
Unsafe	23 (27.1)	39 (45.8)	23 (27.1)	<**0.00**1^a^
Safe	26 (8.3)	157 (49.8)	132 (41.9)	

^a^Pearson Chi Square test was performed; level of significance is at *P* < 0.05.
